# An Integrated Risk-Informed Multicriteria Approach for Determining Optimal Inspection Periods for Protective Sensors

**DOI:** 10.3390/s26010213

**Published:** 2025-12-29

**Authors:** Ricardo J. G. Mateus, Rui Assis, Pedro Carmona Marques, Alexandre D. B. Martins, João C. Antunes Rodrigues, Francisco Silva Pinto

**Affiliations:** RCM2+ Research Centre for Asset Management and Systems Engineering, Lusófona University, Campo Grande, 376, 1749-024 Lisboa, Portugal; rassis46@gmail.com (R.A.); p4803@ulusofona.pt (P.C.M.); p5922@ulusofona.pt (A.D.B.M.); p5942@ulusofona.pt (J.C.A.R.); p6701@ulusofona.pt (F.S.P.)

**Keywords:** sensors, inspection period, maintenance, optimization-simulation, multicriteria, risk, probability management

## Abstract

Equipment failure is the leading cause of industrial operational disruption, with unplanned downtime accounting for up to 11% of manufacturing revenue, highlighting the need for effective proactive maintenance strategies, such as protective sensors that can detect potential failures in critical equipment before a functional failure occurs. However, sensors are also subject to hidden failures themselves, requiring periodic failure-finding inspections. This study proposes a novel integrated multimethodological approach combining discrete event simulation, Monte Carlo, optimization, risk analysis, and multicriteria decision analysis methods to determine the optimal inspection period for protective sensors subject to hidden failures. Unlike traditional single-objective models, this approach evaluates alternative inspection periods based on their risk-informed overall values, considering multiple conflicting key performance indicators, such as maintenance costs and equipment availability. The optimal inspection period is then selected considering uncertainties and the intertemporal, intra-criterion, and inter-criteria preferences of the organization. The approach is demonstrated through a case study at the leading Portuguese electric utility, replacing previous empirical inspection standards that did not consider economic costs and uncertainties, supported by an open, transparent, auditable, and user-friendly decision support system implemented in Microsoft Excel using only built-in functions and modeled based on the principles of probability management. The results identified an optimal inspection period of 90 h, representing a risk-informed compromise distinct from the 120 h interval suggested by cost minimization alone, highlighting the importance of integrating organizational preferences into the decision process. A sensitivity analysis confirmed the robustness of this solution, maintaining validity even as the organizational weight for equipment availability ranged between 35% and 82%. The case study shows that the proposed approach enables the identification of inspection intervals that lead to quantitatively better maintenance cost and availability outcomes compared to empirical inspection standards.

## 1. Introduction

Unplanned downtime costs are estimated at 11% of revenue in the world’s 500 largest manufacturing companies and continue to increase [1]. Unplanned, reactive, or run-to-failure corrective maintenance is no longer viable for managing critical equipment. Instead, systematic preventive maintenance, after a pre-specified operating period, has become the norm. However, in critical environments, these policies have been increasingly replaced by proactive strategies, such as predictive or condition-based maintenance, capable of detecting potential failures before they degrade into functional failures [2].

Protective sensors are often used to continuously gather real-time data on equipment conditions, indicating that a functional failure is imminent or occurring. These online sensors can detect potential failures before escalation, enabling timely interventions that prevent costly functional failures. For instance, it is often possible to take advantage of the degradation of an equipment’s condition to infer that it has started its failure development period (P–F interval), that is, the time interval until a functional failure occurs [2]. Prompt action can then be taken, either manually or automatically (e.g., via an actuator), to shut down the critical equipment, repair it, and mitigate more severe consequences that could result from a functional failure.

However, protective sensors are not immune to failures, making it essential to ensure their proper functioning. Although some failures are evident, many remain hidden and undetected until they cause severe equipment consequences, leading to high reactivation costs and long downtimes. Hidden failures can account for up to half of all failure modes [2], and their late detection is a major source of downtime and reactive maintenance costs across many industrial sectors, further reinforcing the need to optimize inspection intervals.

In this two-unit sensor–equipment system, two scenarios can arise when the equipment fails:

1. The monitoring sensor is well-functioning, detecting, and issuing an early warning of a potential failure in the protected critical equipment promptly, allowing for a prompt equipment shutdown and repair, with minor equipment repair costs and downtime.
2. The monitoring sensor is either ineffective (due to a hidden failure) or non-operational (because it has failed and is still under repair), and a multiple failure occurs in the system, leading to significant consequences in terms of equipment reactivation costs and downtime.

To reduce the probability that the protective sensor is not effectively monitoring and protecting the critical equipment, the former must be inspected periodically to check for the presence of hidden failures [3]. Inspection intervals (failure finding intervals) may be either constant (periodic) or different (sequential or non-periodic). The common practice in the industry is to implement a calendar-based inspection policy with periodic and scheduled inspections, at fixed, equal, and calendar intervals due to its applicability and feasibility.

Naturally, periodic offline inspections incur their own costs. Hence, the trade-off between the consequences of failure-finding inspections in the protective sensor and the consequences of a functional failure in the equipment must be assessed. It is expected that less frequent sensor inspections (and hence longer inspection periods) will decrease the probability of detecting a failure in the equipment and consequently:

1. On the one hand, the expected sensor inspection costs and equipment availability will decrease.
2. On the other hand, the expected equipment reactivation costs and downtime are likely to increase.

Therefore, an optimal interval between periodic inspections is expected to exist, corresponding to the best compromise between the multiple conflicting organizational Key Performance Indicators (*KPIs*) (e.g., minimizing total maintenance costs and maximizing equipment availability) regarding the equipment’s functioning. This compromise can be solved by eliciting the specific preferences of the organization regarding those trade-offs, that is, how much it is willing to lose in one *KPI* (e.g., total costs) to gain in another (e.g., equipment availability).

Unlike previous studies that optimize inspection intervals using a single method (e.g., simulation or risk analysis alone), this work uniquely integrates simulation, optimization, and multicriteria decision analysis into a unified, transparent framework applicable across diverse industrial contexts.

Therefore, the main objective of this article is to present an integrated risk-informed multicriteria value optimization and simulation approach to determine the optimal inspection period for a protective sensor, subject to hidden failures, while monitoring the condition of a critical system. The novelty of this work does not lie in the development of new theoretical algorithms but rather in the coherent integration of established methods (i.e., discrete event simulation, Monte Carlo methods, risk analysis, multicriteria value measurement, and optimization) within a transparent and auditable decision framework. This integrated approach allows organizations to evaluate inspection programs under uncertainty, incorporating their preferences across multiple conflicting *KPIs*. This aim is proposed to be achieved by addressing the following research questions:

1. How to represent and model uncertainties in the occurrence of failures in the equipment and sensors.
2. How to predict the consequences of the occurrence of system failures on multiple *KPIs*, such as total maintenance costs and equipment availability, considering distinct sensor inspection periods, costs, times, and other parameters of the system and the organization.
3. How to model and compare the value and risk of distinct sensor inspection periods, considering their uncertainty and consequences on multiple conflicting organizational *KPIs*.
4. How to find the optimal inspection period considering the previous issues in an integrated and robust approach.
5. How to develop a decision support system that can be easily understood, audited, and used in practice by the organization.

The specific objectives of this article are to answer these research questions through an integrated and generic multimethodological approach and to demonstrate its effectiveness through its application to a real-world case study supported by an open, transparent, auditable, and user-friendly decision support system (DSS).

The remainder of this paper is organized as follows: Section 2 presents a description of the specific proactive maintenance problem tackled by the article, highlighting its multicriteria and stochastic nature, introducing the most important challenges that underpin its analysis and resolution We included a short review of the state of the art on similar published approaches to address the research problem and build the case for the need and contribution of the methodological framework. Section 3 describes the proposed methodology. It starts with a formal definition of the problem, presenting the assumptions and building blocks of the methodology, and detailing the methods, variables, parameters, and equations used in each step of the framework. Section 4 illustrates the application of the approach through a case study supported by a DSS. Section 5 discusses the main contributions to the research literature and practice and identifies its strengths and limitations. Section 6 concludes the paper and suggests potential avenues for future research.

## 2. State of the Art and Research Gap

Industrial systems degrade over time, leading to system failures that result in increased costs, unexpected machine unavailability, and quality and safety issues. While maintenance has historically been regarded as a reactive necessity, it is now recognized as a critical activity of asset management aimed at improving organizational efficiency, reliability, and profitability [4].

Within the context of Industry 4.0 and 5.0, traditional maintenance approaches—such as reactive and preventive maintenance—are progressively being replaced by more proactive strategies based on condition monitoring and predictive analytics, which aim to reduce maintenance costs and improve equipment availability and reliability [5,6].

A proactive maintenance system typically comprises two main types of components: (i) critical entities that perform the system’s fundamental function and (ii) auxiliary entities that provide information, redundancy, and/or protection [7,8]. Protective components serve to mitigate the consequences of the protected critical equipment failure, which would otherwise be significantly more severe [2].

Proactive maintenance relies heavily on sensor data to monitor equipment condition, enabling early anomaly detection, performance optimization, increased system reliability, and the dynamic scheduling of maintenance interventions before failures occur. Predictive maintenance combines historical sensor data with predictive models based on statistical, forecasting, or artificial intelligence methods to estimate the likelihood and timing of future failures [9,10,11,12].

Reliability Centered Maintenance (RCM) constitutes a structured and widely adopted proactive framework that prioritizes critical assets and enables the development of customized maintenance plans, which reduce downtime and operational disruptions, extend equipment lifespan, and promote more efficient, reliable, sustainable, and cost-effective operations, ultimately improving product quality and customer satisfaction. RCM’s plans track equipment performance through a combination of condition-based monitoring of real-time sensor data and predictive analytics to anticipate failures and schedule preventive maintenance interventions accordingly before failures escalate into major consequences.

Nevertheless, most maintenance decision studies assume that the monitoring sensor is perfect, that is, its information signal is accurate to the actual state of the equipment, overlooking the compounded effect of sensor unreliability due to the presence of hidden failures. Faulty sensors that produce erroneous signals lead to incorrect maintenance decisions [13]. Research on the maintenance of a critical system with an unreliable protective system is limited [14]. Hence, more research should be conducted to study the effects of imperfect condition monitoring on optimal dynamic maintenance decisions, namely, new models and methodologies that determine the optimal inspection maintenance schedule based on predicted failures of the sensor and equipment [15].

A numerical method for determining the optimal inspection calendar of critical equipment that fails according to a Weibull probability distribution was proposed, considering the minimization of total maintenance costs and the time interval between a potential failure and a functional failure (the so-called P–F interval, failure development period, or warning period) [16]. The Weibull distribution is widely used in reliability analysis because of its versatility in describing either failure (event) times or the lifetime of systems or their components subject to random failures or degradation phenomena (e.g., wear-out, corrosion, erosion, and fatigue).

Despite the advances in RCM, the current inspection maintenance optimization best practice still often relies on simplified closed-form formulas for the problem of finding the optimal inspection scheduling for two-unit systems [17]. For instance, to determine the optimal inspection period ∆T (failure-finding interval) for a single independent protective sensor, the RCM standard guide standard suggests a simple formula, expressed in Equation (1), based only on the Mean Time Between Failures (MTBF) of the equipment, the sensor, and the (multiple-failure) system [2]:

(1) $$∆T=\frac{2\;\times \;{MTBF}_{sensor}\;\times \;{MTBF}_{equipment}}{{MTBF}_{system}}.$$

While formulas provide an intuitive approach to certain types of problems, they are often not suitable for defining the optimal inspection period for two-unit systems because of the computational complexities, dependencies, nonlinear relationships, uncertainties, and dynamic nature of such systems. Alternative methods, such as numerical, simulation, optimization, and multicriteria decision analysis methods, offer greater flexibility and can better accommodate the intricate relationships and real-world complexities inherent in these systems.

Additionally, most existing solutions focus only on a single optimization objective, limiting their applicability in real-world industrial practice [17,18]. For instance, Equation (1) does not account for related maintenance costs or other relevant organizational objectives. However, in maintenance optimization, there are often multiple and typically conflicting objectives, including maintenance cost minimization and equipment availability maximization, among others [6]. Although there are articles that use multicriteria decision analysis methodologies to select the best inspection period [19,20], we are not aware of any that specifically addresses the research problem of this article.

Ref. [18] conducted a systematic literature review on industrial maintenance decision-making to identify the efforts required for future work development and new integrated methodologies, concluding that the following difficulties and challenges are still present in current decision-making models and methodologies:

1. Lack of qualitative/quantitative integration between the correct treatment data (e.g., costs, probability of failures, equipment condition, and risk analysis) and the preferences of the decision maker regarding multiple objectives.
2. Definition of meaningful *KPIs* and creation of methods for transforming information into actionable knowledge for proactive maintenance decisions.
3. The integration of methodologies in which decision-making modeling is continuously fed using reliable results from a specific step into the models at another stage.

Despite these developments, existing studies still present important limitations. Many rely on single-objective optimization, typically cost minimization or availability maximization, and therefore fail to represent the conflicting nature of real industrial *KPIs*. Others assume perfect monitoring sensors, disregarding the impact of hidden failures on maintenance decisions. Furthermore, analytical closed-form formulas, while convenient, oversimplify system interactions and cannot adequately capture uncertainty or dynamic behavior. In contrast, the present work combines risk-informed modeling, discrete-event simulation, optimization techniques, and multicriteria decision analysis into a unified and transparent framework that explicitly accounts for sensor unreliability and multiple organizational objectives.

This literature review clearly highlights the need for developing risk-informed optimization-simulation models that support multicriteria decision-making for determining the optimal inspection scheduling of protective sensors monitoring critical equipment, accounting for their failure uncertainties and interdependence dynamics.

## 3. Integrated Methodological Approach

The target maintenance system, S, consists of two units: (a) an equipment, e (or one of its components), providing a critical function to the organization, which can fail and needs protection; (b) a sensor, s, providing a continuous online protective function to equipment, e, triggering an alarm when abnormal conditions are detected and, therefore, also subject to its own failures.

The proposed maintenance policy involves a periodic inspection of the protective sensor. Inspections occur at fixed and predetermined intervals ∆T, resulting in a calendar schedule where each inspection i is performed at time $t_{i}$.

Two system-level failure scenarios can occur when the protected equipment fails:

1. Normal detected failure: The sensor is operational and promptly identifies a potential failure fe in the equipment, which is immediately halted and restored within a repair time ∆tre and at unit cost cre.
2. Multiple (undetected) failures in the system S: The sensor is ineffective or unavailable, and an equipment failure results in a significantly longer reactivation period, ∆trS and higher unit cost, crS.

The following modeling assumptions apply:

1. Failure times of equipment and sensors are statistically independent.
2. Failure times follow a three-parameter Weibull probability distribution.
3. Sensor and equipment inspections are assumed to be perfect, revealing their true operational states. This assumption simplifies the initial model. Incorporating inspection errors (e.g., false negatives or false positives) constitutes a logical extension and would generally lead to shorter optimal inspection intervals to maintain the desired system’s reliability and equipment availability.
4. Inspection duration is assumed to be negligible. In practical applications, sensor inspections may require a brief equipment shutdown. The model can be easily refined to account for this by adding a fixed downtime period to each inspection without affecting the overall simulation logic or methodology.
5. Each sensor inspection incurs a fixed cost.
6. Repairs begin immediately upon failure detection.
7. Each unit is restored to its initial (as-new) condition after each repair or reactivation.
8. Repair and reactivation durations and costs are fixed constants.
9. A constant annual discount rate is applied.

**Figure 1:**
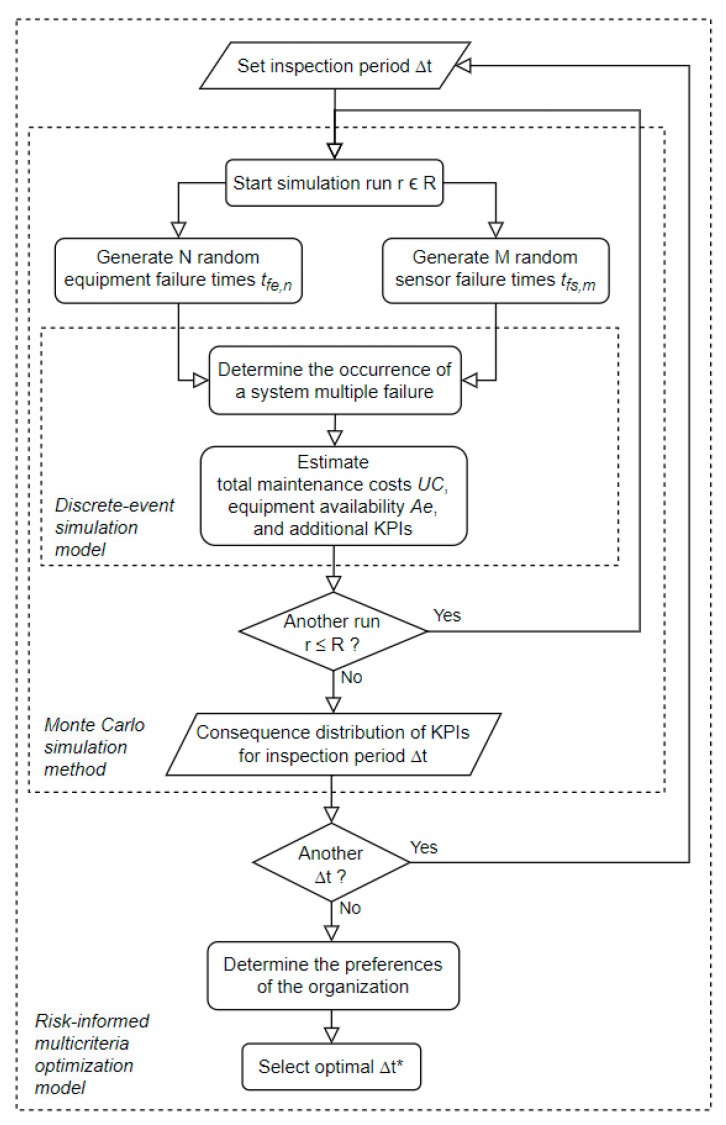
Diagram logic of the methodology to select the optimal ∆T*.

The optimal inspection period ∆T* is determined through a four-step methodology (see Figure 1):

1. Random Failure Time Generation: Generate failure times $t_{fe}$ and $t_{fs}$ according to Weibull distributions to model the uncertainty in the occurrence of equipment and sensor failures.
2. Discrete-Event Simulation: Execute a run until a multiple failure occurs and estimate consequences on *KPIs* (e.g., total maintenance costs, equipment availability) as a function of ∆T and other relevant input parameters.
3. Monte Carlo Simulation: Repeat the random sampling procedure (in Step 1) to generate the distributions of *KPI* consequences (from Step 2) for each ∆T.
4. Inspection Period Optimization: Select the ∆T* that maximizes the risk-adjusted overall value using a risk-informed multicriteria decision analysis methodology.

The candidate inspection periods are evaluated over a discrete grid defined by a step size. In practice, the step size should be chosen small enough to ensure that the difference in overall value between adjacent ∆T periods is negligible in the face of the uncertainty of the simulation results A practical guideline is to set the step size to no more than 5–10% of the expected inspection interval, and to perform iterative refinement around the candidate optimum to confirm the robustness of the solution.

The variables, parameters, equations, and methods used in this methodological sequence are detailed next.

### 3.1. Random Failure Times Generation for the Sensor and Equipment

**Figure 2:**
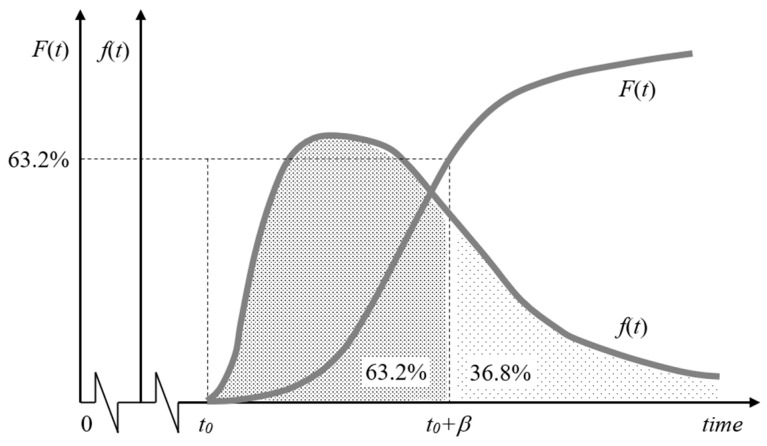
Probability density *f*(*t*) and cumulative distribution *F*(*t*) functions of the Weibull distribution.

Uncertainty in the time to failure of the equipment and sensor s is modeled using a three-parameter Weibull distribution (Figure 2).

The probability density function *f*(*t*) of each unit is represented by Equation (2):

(2) $$f\left(t\right)=\frac{\alpha }{\beta }\cdot {\left[\frac{t-t_{0}}{\beta }\right]}^{\alpha -1}\cdot {exp}^{-{\left(\frac{t-t_{0}}{\beta }\right)}^{\alpha }}\mathrm{with}\;t\geq t_{0},\;\alpha >0,\;\mathrm{and}\;\beta >0$$

where t represents the time of failure, exp represents the base of natural logarithms (approximately 2.7183), $t_{0}$ is a location (or threshold) parameter that defines the lowest value that t can take and allowing to offset the distribution by the repair time tr of the unit that it re-enters into service after a failure (and restoring its initial capability as new), β is a positive scale (or spread) parameter defining the time t at which 63.2% of the units have failed and representing the characteristic life of the unit, and α is a positive shape (or slope) parameter reflecting the failure degradation mechanism of the unit such that it is one if the failure rate is random and either greater or less than one if that rate increases (e.g., early or rapid wear-out) or decreases (e.g., infant mortality) over time respectively. Parameters α and β should be determined by fitting empirical data from experiments, expert analysis, or theoretical values from the respective suppliers or from reliability data sources (e.g., OREDA database [21]).

The cumulative distribution function of the Weibull distribution $F\left(t\right)$ can be derived by integrating the density function $f\left(t\right)$ between tr (i.e., $t_{0}$) and t resulting in Equation (3):

(3) $$F\left(t\right)=1-{exp}^{-{\left(\frac{t-tr}{\beta }\right)}^{\alpha }}.$$

By inverting Equation (3) and replacing $F\left(t\right)$ by a uniform continuous random variable rnd between 0 and 1 results in Equation (4), which can be used to generate the random failure time t according to Equation (3) by randomly sampling values from rnd:

(4) $$t=tr+\beta \cdot {\left[\ln\left(\frac{1}{1-rnd}\right)\right]}^{\frac{1}{\alpha }}.$$

**Figure 3:**
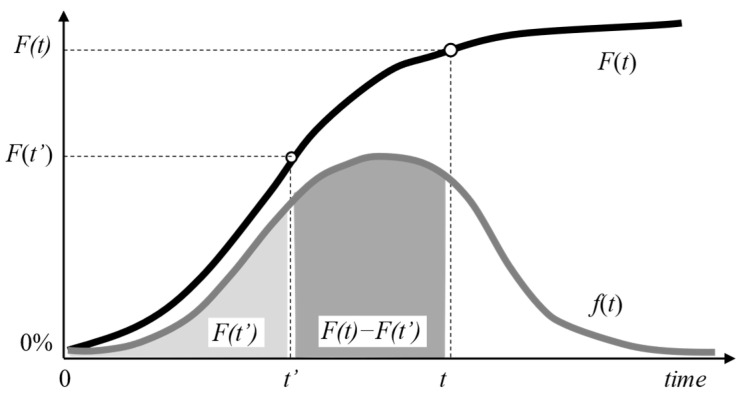
Conditional probability of failure $F\left(t-t^{\prime }|t^{\prime }\right)$ in time $t-t^{\prime }$ having already survived until time $t^{\prime }$.

If the unit has already survived up to time $t^{\prime }$, the respective probability of a failure can be determined by the conditional probability of failure $F\left(t-t^{\prime }|t^{\prime }\right)$, given by Equation (5), which computes the probability that the unit will fail in time period t given that it has already survived time $t^{\prime }$ (see Figure 3):

(5) $$F\left(t-t^{\prime }|t^{\prime }\right)=\frac{F\left(t\right)-F\left(t^{\prime }\right)}{1-F\left(t^{\prime }\right)}.$$

The inverse of Equation (5) results in Equation (6), where $F\left(t^{\prime }\right)$ is computed by Equation (3). Hence, rather than Equation (4), Equation (6) must be used for generating the first random failure time t for a unit that has already accumulated some service time $t^{\prime }$ at the start of the simulation:

(6) $$t=\beta \cdot {\left[\ln\left(\frac{1}{1-\left[F\left(t^{\prime }\right)+rnd\cdot \left[1-F\left(t^{\prime }\right)\right]\right]}\right)\right]}^{\frac{1}{\alpha }}-t^{\prime }.$$

After the first failure and subsequent repair, the unit is assumed to restore its initial condition as new. The next random failure time t is afterwards determined using Equation (4).

The repair time tr after a failure event is determined differently for the sensor and the equipment.

The sensor can have at most one failure within each inspection period, since it can only be detected at the time $t_{i}$ of the next inspection i. Time $t_{i}$ can be determined by the multiplication of ∆T by the rounded-up integer of the division of the sensor failure time $t_{fs}$ by ∆T. The sensor is then started repairing at time $t_{i}$ for a repair time ∆trs until it has been repaired at time ${tr}_{fs}$, as expressed by Equation (7):

(7) $${tr}_{fs}=\left\lceil \frac{t_{fs}}{∆T}\right\rceil \cdot ∆T+∆trs.$$

The equipment can have multiple failures within each inspection period, since the sensor can promptly detect any equipment failure. The equipment is then immediately started repairing at the equipment failure time $t_{fe}$ for a repair time ∆tre until it has been repaired at time ${tr}_{fe}$, as expressed by Equation (8):

(8) $${tr}_{fe}=t_{fe}+∆tre.$$

### 3.2. Discrete-Event Simulation Model

A discrete-event simulation replicates the system until a multiple failure occurs, allowing the estimation of consequences for each ∆T using the previously generated random failure times, and other relevant input parameters.

#### 3.2.1. Determining the Occurrence of a Multiple Failure in the System

**Figure 4:**
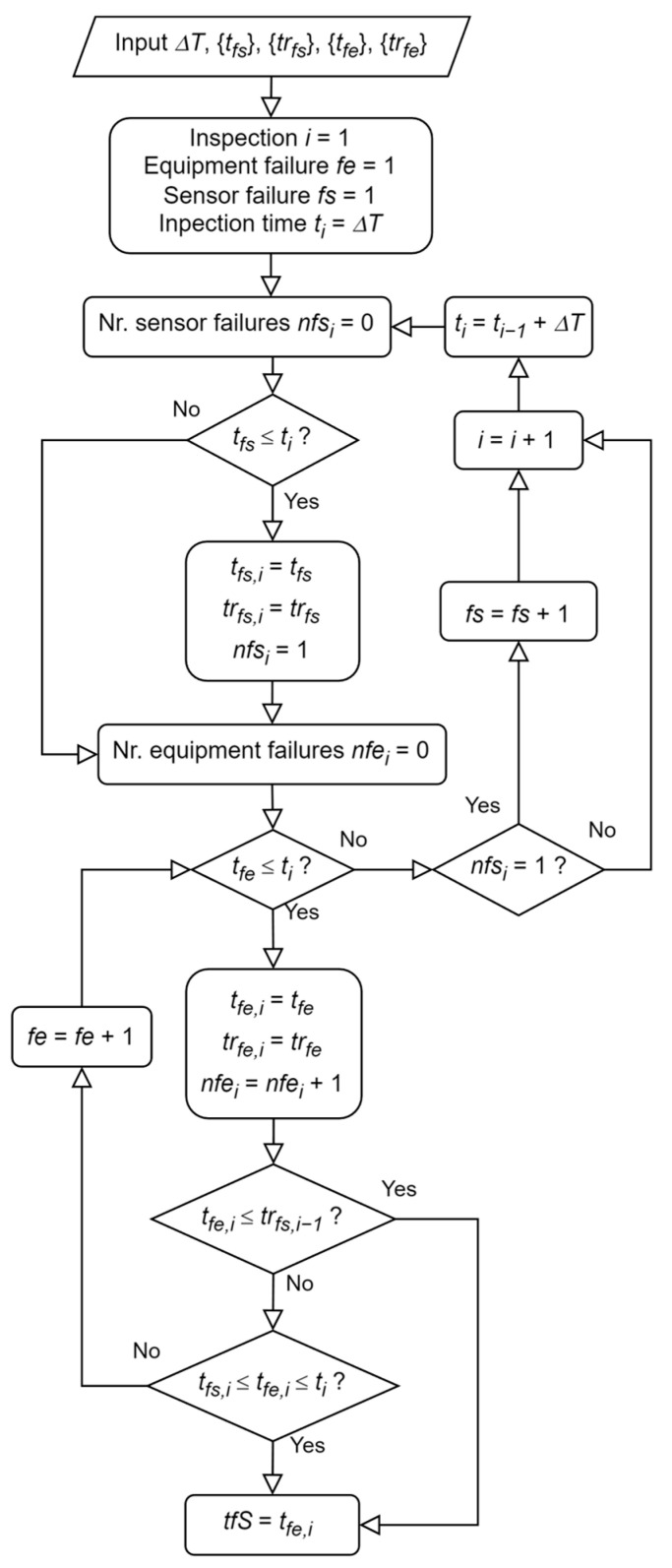
Discrete-event simulation model to find the system multiple failure time.

A multiple failure occurs at time tfS when an equipment failure occurs within an interval in which the sensor is unavailable, that is, between a sensor failure time $t_{fs}$ and the time ${tr}_{fs}$ until it is repaired. Figure 4 presents the procedure to find tfS along with the number of equipment failures ${nfe}_{i}$ and sensor failures ${nfs}_{i}$ occurring within each period ${∆t}_{i}={[t}_{i-1};t_{i\;}]$ between each inspection.

#### 3.2.2. Determining Total Maintenance Costs Until the Occurrence of a Multiple Failure

Total maintenance costs are computed based on the sum of the inspection costs, sensor repair costs, equipment repair costs, and system reactivation costs incurred during time horizon H=tfS+∆trS, that is, up to the time when the equipment is reactivated (∆trS) following a multiple failure (tfS).

The organizational time preference to incur costs later than sooner is modeled by discounting them with a discount rate d, allowing the conversion of any future cost $F_{t}$ into a commensurate amount representing its equivalent present cost P as expressed in Equation (9):

(9) $$P=\frac{F_{t}}{{\left(1+d\right)}^{t}}\;.$$

Alternative time series of equivalent present cash outflows can then be meaningfully added up and compared. Discount rates should be set by the organization based on the opportunity costs associated with both its capital cost and the investment risk premium, that is, the average risk-adjusted return on investment that the organization can obtain from alternative investments.

Organizations typically offer discount rates annually. Maintenance events usually occur in shorter time periods though. Therefore, the annual discount rate could be converted into an equivalent rate $d^{\prime }$ expressed in a more convenient time unit (e.g., minutes, hours, weeks) using Equation (10), where nu is the number of time units u (e.g., hours) within one year (e.g., 365 × 24 = 8760 h):

(10) $$d^{\prime }={\left(1+d\right)}^{\frac{1}{nu}}-1.$$

Total sensor inspection present costs, TPi, incurred until a multiple failure occurs (in each simulation run) are determined by Equation (11):

(11) $$TPi=\sum_{i}^{I}\frac{ci}{{\left(1+d^{\prime }\right)}^{t_{i}}}$$

where I is the number of inspections i, ci is the sensor inspection unit cost, and $t_{i}$ is the time of each inspection i. Total sensor repair present costs TPrs are determined by Equation (12):

(12) $$TPrs=\sum_{i}^{I}\frac{crs\cdot {nfs}_{i}}{{\left(1+d^{\prime }\right)}^{t_{i}}}+\frac{crs}{{\left(1+d^{\prime }\right)}^{tfS}}$$

where crs is the sensor repair unit cost, ${nfs}_{i}$ is the number of sensor failures (either one or null) within the respective $∆t_{i}$, $t_{i}$ is the time of inspection i, and tfS is the system failure time. Note that after the last inspection I there is always an extra sensor repair cost at the time tfS since the sensor failure is detected only then. Total equipment repair present costs TPre are determined using Equation (13):

(13) $$TPre=\sum_{i}^{I}\sum_{fe}^{{nfe}_{i}}\frac{cre}{{\left(1+d^{\prime }\right)}^{t_{fe,i}}}+\sum_{\left(fe|i=I+1\right)}^{{nfe}_{i}-1}\frac{cre}{{\left(1+d^{\prime }\right)}^{t_{fe,i}}}$$

where cre is the equipment repair unit cost, ${nfe}_{i}$ is the number of equipment failures within the respective $∆t_{i}$, and $t_{fe,i}$ is the time of failure fe within the respective $∆t_{i}$. Note that after the last inspection I there might be additional equipment repair costs for the first equipment failures fe before the system fails. Finally, total equipment (or system) reactivation present costs, TPrS, are determined using Equation (14):

(14) $$TPrS=\frac{crS}{{\left(1+d^{\prime }\right)}^{tfS}}$$

where crS is the equipment (or system) reactivation unit cost. Since the previous total present costs, TP, are determined based on different time horizons for each simulation run, it is necessary to convert them into a series of (present) costs UC made at equal (uniform) time intervals to compare them. The equivalent uniform cost can be computed by the product of TP by two factors: a uniform time interval tu (e.g., 100 h) and a conversion factor fc as expressed in Equation (15):

(15) $$fc=\frac{{d^{\prime }\left(1+d^{\prime }\right)}^{H}}{{\left(1+d^{\prime }\right)}^{H}-1}.$$

Uniform system total costs UC for each simulation run can then be computed as expressed in Equation (16):

(16) $$UC=fc\times tu\times \left(TPi+TPrs+TPre+TPrS\right).$$

#### 3.2.3. Determining Other *KPIs* Until the Occurrence of a Multiple Failure

Additional *KPIs* can be computed as required. A common *KPI* in maintenance is the equipment availability Ae, that is, the proportion of time the equipment can perform its intended function, as expressed in Equation (17):

(17) $$Ae=\frac{total\;time-unavailable\;time}{total\;time}=1-\frac{\left[\left(Nfe-1\right)\Delta tre+\Delta trS\right]}{tfS+\Delta trS}.$$

Other common metrics such as Mean Time To Failure (MTTF), Mean Time Between Failures (MTBF), or Mean Time to Repair (MTTR) may be used depending on the organization’s objective, but equipment availability is retained as the primary reliability *KPI* in this study.

### 3.3. Generating Distributions of Consequences on KPIs for Each ΔT Through a Monte Carlo Method

Uncertainty in the equipment and sensor failure behavior is propagated using Monte Carlo simulation. For each ΔT, the discrete-event simulation is repeated R runs, generating a distribution of *KPI* consequences.

For each *KPI*, expected values and two-tailed confidence interval for a confidence level 1−α are computed using Equation (18):

(18) $$CI=\bar{x}\pm z_{1-\frac{\alpha }{2}}\times \frac{s}{\sqrt{R}}$$

where s is the sample standard deviation and $z_{1-\frac{\alpha }{2}}$ is the *z*-score from the standard normal distribution corresponding to a cumulative probability of $1-\frac{\alpha }{2}$. The precision, stability, and reliability of the results increase with the number of runs set for the Monte Carlo simulation at the cost of higher computation demands in terms of memory and running time. The choice of the right trade-off between precision and performance time is context-dependent and should be determined by the organization based on the specific application.

### 3.4. Determining the Optimal Inspection Period ΔT* Through a Risk-Informed Multicriteria Value Measurement Method

Organizations often face multiple conflicting performance objectives, including costs, availability or reliability, product quality or throughput, customer service, safety, and environment. Without loss of generality to further objectives, the optimal inspection period ΔT* will be determined in this case study for only two of the most used conflicting objectives in maintenance problems: minimizing total maintenance costs (Equation (16)) and maximizing equipment availability (Equation (17)). Maintenance cost and equipment availability were selected because they are among the most widely adopted and operationally critical *KPIs* in industrial maintenance. The framework remains fully extensible and can incorporate additional objectives.

Conflicting objectives involve a trade-off: improving the performance on one *KPI* can only be achieved by compromising the performance on the other, and vice versa. However, organizations must still choose the best ΔT* between conflicting objectives. Multicriteria value measurement methods (MCVM) provide a solution to this conundrum by modeling the intra-criterion and inter-criterion preferences of the organization regarding multiple objectives.

Intra-criterion preferences on each *KPI* can be defined by value functions $v\left(KPI\right)$ representing how much the organization values marginal consequences on the respective *KPI*, and inter-criteria preferences by weights $w_{KPI}$, representing how much the organization is willing to lose on one *KPI* to increase on another.

The optimal inspection period, ΔT*, is then the one that maximizes the overall value $V\left(\Delta T\right)$ of each potential alternative computed by the additive multicriteria value model expressed in Equation (19):

(19) $$\Delta T*=\arg\underset{\Delta t}{\max}V\left(\Delta T\right)=\arg\underset{\Delta t}{\max}\left(\sum_{KPI}w_{KPI}\times v\left(KPI\left(\Delta T\right)\right)\right)\;and\;\sum_{KPI}w_{KPI}=1.$$

The MCVM literature describes various well-established techniques for constructing a value function for continuous *KPIs* based on the elicitation of preferences from the decision-maker, namely, the bisection method [22], direct rating [23], or MACBETH [24]. The bisection method provides a simple and sound protocol for assessing either linear or exponential functions on each *KPI*: (a) it starts by scoring the worst (or a bad) consequence ${KPI}^{-}$ with a value score $v\left({KPI}^{-}\right)=0$, and the best (or a good) consequence ${KPI}^{+}$ with a value score $v\left({KPI}^{+}\right)=100$; (b) it then asks the decision-maker to identify the intermediate consequence ${KPI}^{\prime }$ that makes them indifferent between either improving ${KPI}^{-}$ to ${KPI}^{\prime }$ or improving ${KPI}^{\prime }$ to ${KPI}^{+}$; and (c) it finally fits a function that passes through the points $\left({KPI}^{-},0\right),\;\left({KPI}^{\prime },50\right),\;and\left({KPI}^{+},100\right)$. If the resulting function is linear, its closed-form expression corresponds to Equation (20). Otherwise, it can be fitted through the exponential function respecting the delta property, expressed in Equation (21):

(20) $$v\left(KPI\right)=100\times \frac{KPI-{KPI}^{-}}{{KPI}^{+}-{KPI}^{-}}$$

(21) $$v\left(KPI\right)=100\times \frac{1-{exp}^{-\frac{KPI-{KPI}^{-}}{\rho }}}{1-{exp}^{-\frac{{KPI}^{+}-{KPI}^{-}}{\rho }}}.$$

The MCVM literature also describes sound procedures for assessing the weights associated with the *KPIs* based on the elicitation of preferences from the decision-maker, namely the trade-off procedure [25], swing weighting [23], and MACBETH [26]. The trade-off procedure provides a practical and sound questioning protocol for determining the weights associated with the *KPIs* in this setting because decision-makers usually find it easier to assess non-monetary *KPIs* against a monetary *KPI*, such as the total maintenance costs UC. This procedure comprises the following: (a) for each KPI\{UC} ask the decision-maker to identify the total maintenance cost ${UC}^{KPI}$ that makes him (or her) indifferent between a fictitious alternative A defined by the consequences ${UC}^{-}$ and ${KPI}^{+}$ and a fictitious alternative B defined by ${UC}^{KPI}$ and ${KPI}^{-}$; (b) compute the weights $w_{KPI}=\frac{v\left({UC}^{KPI}\right)}{\sum_{KPI}v\left({UC}^{KPI}\right)}$ where $v\left({UC}^{UC}\right)=100$.

A notable special case occurs when $v\left(UC\right)$ is assessed to be linear, where weights $w_{KPI}$ can be more simply determined by: (a) asking the decision-maker to assess how much ${UC}^{KPI}$ is he (or she) willing to pay for improving each *KPI* from ${KPI}^{-}$ to ${KPI}^{+}$; (b) computing the weight $w_{KPI}=\frac{{UC}^{KPI}}{\sum_{KPI}{UC}^{KPI}}$. Additionally, in this special case, steps (b) and (c) of the previously described bisection method can be rather performed by: (b) asking the decision-maker to assess how much ${UC}_{KPI}^{\prime }$ is he (or she) willing to pay for improving each *KPI* from ${KPI}^{-}$ to a given intermediate consequence ${KPI}^{\prime }$; (c) fitting a function that passes through the points $\left({KPI}^{-},0\right),\;\left({KPI}^{\prime },100\times \frac{{UC}_{KPI}^{\prime }}{{UC}_{KPI}^{+}}\right),\;and\left({KPI}^{+},100\right)$.

Decision alternatives comprise a set of evenly distributed inspection periods ΔT. Uncertainty in sensor and equipment failures propagates through the discrete-event simulation model and the additive multicriteria value model, resulting in an uncertain overall value $V\left(\Delta T\right)$ for each ΔT. This uncertainty can be represented as a risk profile encapsulating the range of possible overall values $V\left(\Delta T\right)$ and their associated probabilities. If the cumulative risk profile of one alternative ΔT lies entirely to the right of another, the former stochastically dominates the latter [27]. When a single ΔT stochastically dominates all others, it is deemed optimal. Otherwise, in contexts where organizations face many similar decisions, a risk-neutral preference is generally the most appropriate risk attitude. Accordingly, the optimal inspection period ΔT* should correspond to the ΔT with the highest expected value, $\bar{V}\left(\Delta T\right)$, as defined by the additive multicriteria value model in Equation (19).

Any potential optimal decision must always be stress-tested against the range of significant uncertainties identified during the construction of the respective decision support model, namely, the parameters of the additive multicriteria value model. The sensitivity analysis can inform the organization on the robustness of the preliminary decision to select a given optimal inspection period against changes in the *KPIs*’ weights.

## 4. Case Study

**Figure 5:**
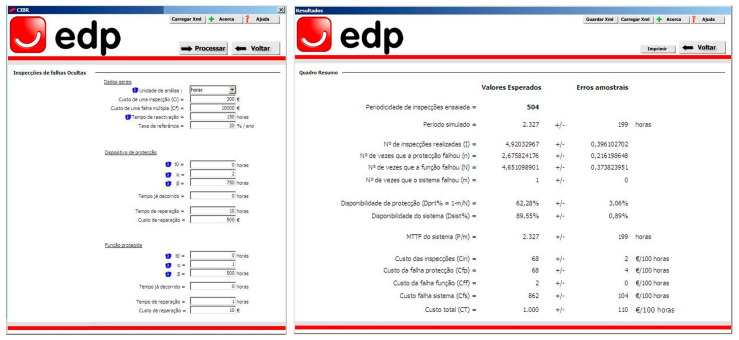
DSS was implemented in a Portuguese electric utility organization.

A case study of the methodology described in Section 3 is presented next to demonstrate and validate its application in a real-world decision context. The case study will be illustrated using specific inputs but is generalizable to any other scenarios. Although the approach has been implemented in Portuguese organizations through decision support systems specifically developed for user interaction (see Figure 5), the article proceeds by presenting a prototype Excel application, which can be downloaded from the Supplementary File, for reproducibility, reuse, clarity, transparency, and the reader’s convenience. The proposed methodological approach is independent of the software platform: all steps (random failure generation, discrete event simulation, Monte Carlo analysis, multicriteria methods, risk analysis) can be implemented in alternative environments (e.g., Python 3.0, R 4.2, MATLAB R2022b). The Excel-based DSS should therefore be interpreted as a pedagogical and technology transfer tool rather than a methodological constraint. Additionally, the use of probability management principles ensures that all stochastic elements remain fully traceable and reproducible.

### 4.1. Setting Input Parameters and Reference Units

**Figure 6:**
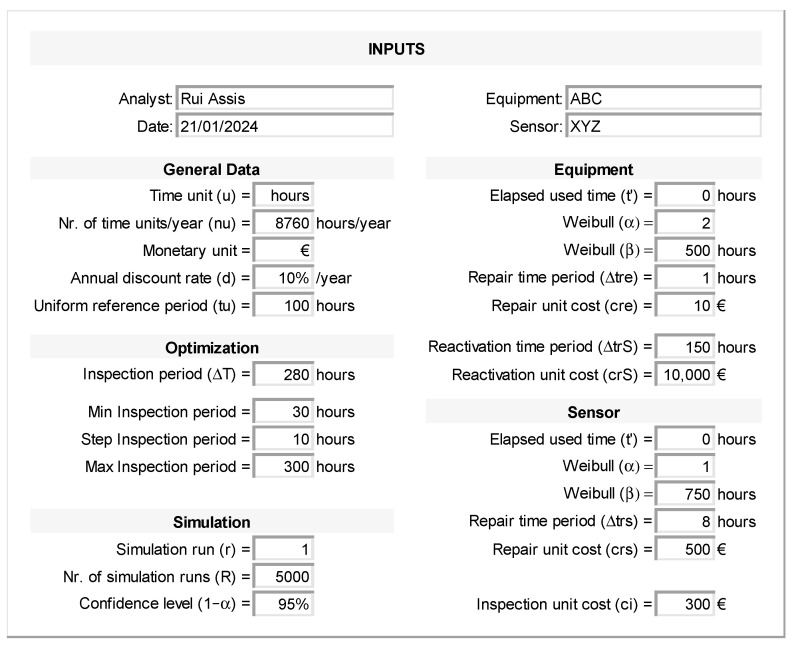
User inputs interface (sheet “Inputs”).

Figure 6 presents the inputs interface (sheet “Inputs”) where the user can set all the input parameters, including their reference units. Symbols used in the notation of Section 3 are depicted within parentheses. All the remaining sheets are fed in from the values filled in this interface. Time and monetary units can be changed. The general data section also allows setting the annual discount rate d of the organization and a uniform reference period tu of the convenience. The optimization and simulation sections allow setting a particular inspection period ΔT (e.g., 280 h) and simulation run r (e.g., one) to verify the respective outcomes in the other interfaces (sheets). The optimization section also allows defining a set of evenly spaced inspection periods from a minimum of 30 to a maximum of 300 and a step of 10. The initial step should be small enough so that differences in the overall value *V*(∆*T*) between adjacent intervals are negligible compared to the result uncertainty. An iterative refinement with a finer step around a candidate optimum is recommended to confirm robustness. The total number of runs R and a particular confidence level (1−α) associated with the desired margin of error of the expected values can be set in the simulation section. The equipment and sensor sections allow filling in the respective parameters $t^{\prime }$, α, and β of the Weibull distribution representing their random failure time, repair time periods Δtre and Δtrs, and repair unit costs cre and crs. The unit cost ci of each inspection can be set in the sensor section, while the equipment’s reactivation unit cost, crS, and reactivation time, ΔtrS can be set in the equipment section.

### 4.2. Generation of Random Failure Times for the Sensor and Equipment

**Figure 7:**
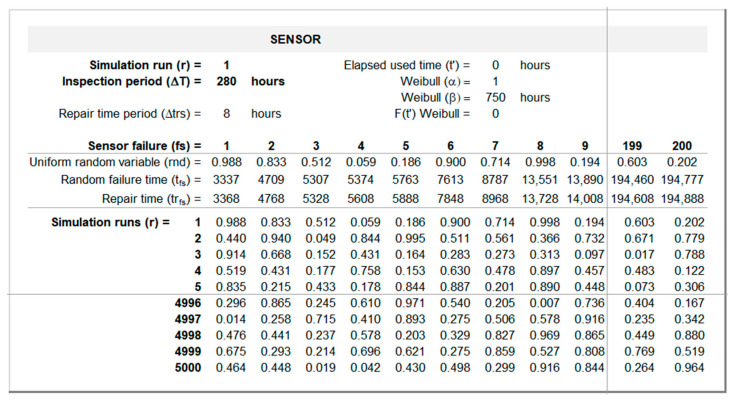
Sensor random failure times interface (sheet “Sensor”).

**Figure 8:**
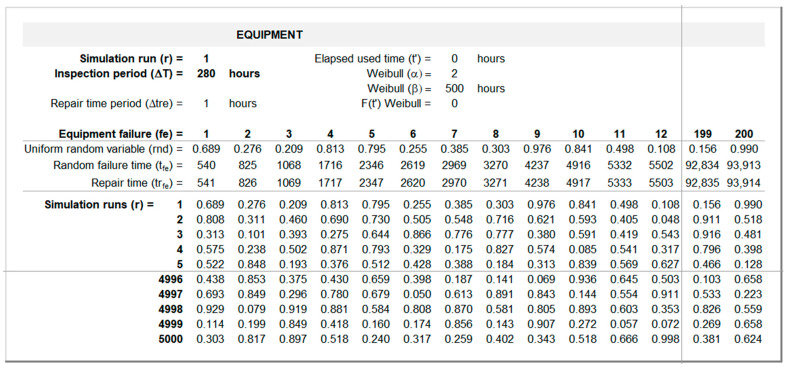
Equipment random failure interface (sheet “Equipment”).

Figure 7 and Figure 8 show the generated random failure times for the sensor and equipment. The top panels use data from the “Input” sheet (Figure 6) to compute these values for each simulation run and inspection period. The bottom panels present a set of pre-generated two hundred uniform random real numbers between 0 and 1 using the Excel function RAND() and then frozen and stored for each simulation run.

These streams are called Stochastic Information Packets (SIPs) by the discipline of probability management [28], allowing auditing, transparency, and understanding of the computations carried out in each simulation run by the user.

Figure 7 illustrates how $t_{fs}$ and ${tr}_{fs}$ are computed based on these data for r = 1 and ΔT = 280 h. The first random failure time is computed through Equation (6) with $F\left(t^{\prime }\right)$ being determined by Equation (3). Repair times are determined by Equation (7). The remaining random failure times are computed through Equation (4).

Figure 8 illustrates how $t_{fe}$ and ${tr}_{fe}$ are computed for r = 1 and ΔT = 280 h. The first random failure time is computed through Equation (6), with $F\left(t^{\prime }\right)$ being determined by Equation (3). Repair times are determined by Equation (8). The remaining random failure times are computed through Equation (4).

### 4.3. Determining the System’s Multiple Failure Time and Consequences on the KPIs

**Figure 9:**
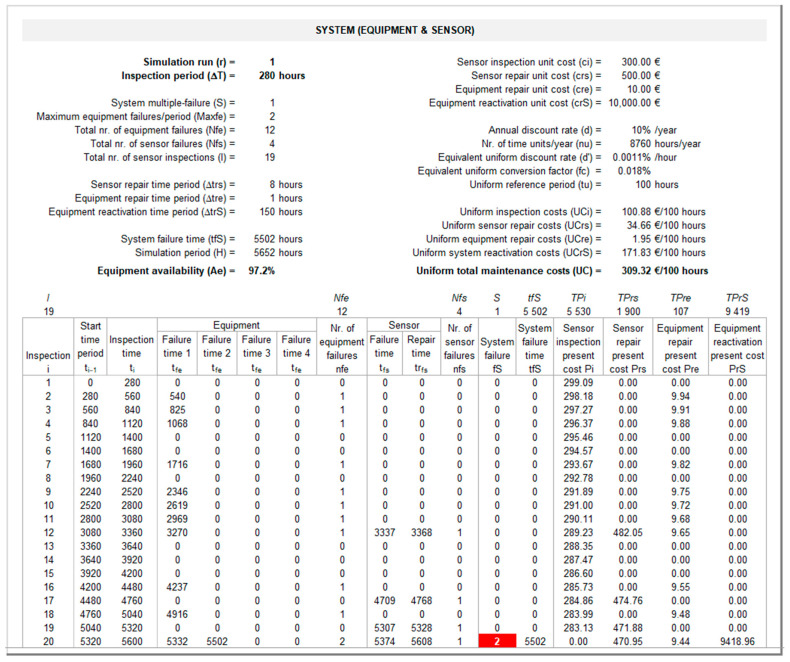
System (multiple) failure and *KPIs* interface (sheet “System”).

The output interface for the discrete-event simulation model and resulting consequences on the *KPIs* is presented in Figure 9 (sheet “System”). The bottom panel of this interface presents the results of the discrete-event simulation model described in Section 3.2.1 until a multiple failure occurs in the system at time tfS. Figure 9 illustrates the computation of tfS for r = 1 and ΔT = 280 h. After 12 equipment failures (Nfe) and four sensor failures (Nfs), a system failure occurs at time tfS = 5502 h, after the 19th and before the 20th inspection is conducted. During this period, the equipment fails a first time at $t_{fe}$ = 5322 h, but since the sensor is operational, the equipment is promptly repaired in Δtre = 1 h and is back working at ${tr}_{fe}$ = 5323 h; later on, the equipment fails again at time $t_{fe}$ = 5502 h, but since at this time the sensor is not operational (from $t_{fs}$ = 5374 to ${tr}_{fs}$ = 5608 h), a multiple failure occurs at tfS = 5502 h. The last four columns from the bottom panel in Figure 9 show the total present costs associated with sensor inspections (TPi), sensor repairs (TPrs), equipment repairs (TPre), and equipment reactivation (TPrS) computed as described in Section 3.2.2 using Equations (10)–(14). The top panel in Figure 9 presents the relevant inputs, and the resulting consequences on the two organizational *KPIs*. Total maintenance costs are measured through the respective uniform total maintenance costs UC, determined using Equations (15) and (16) described in the last paragraphs of Section 3.2.2, considering a uniform reference period tu equal to 100 h. Equipment availability Ae is computed using Equation (17) described in Section 3.2.3.

### 4.4. Estimating the Distribution of Consequences of Distinct Inspection Periods on KPIs Through Monte Carlo Simulation

**Figure 10:**
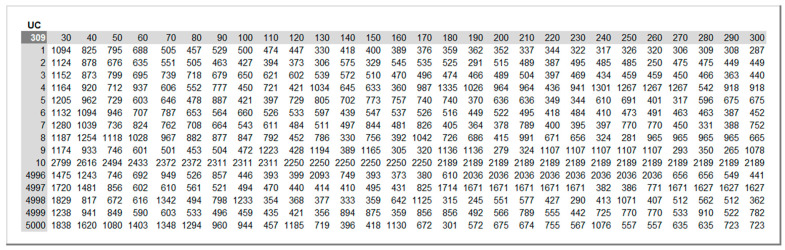
Total maintenance costs UC across 5000 runs for inspection periods ΔT = 30 to 300 h.

**Figure 11:**
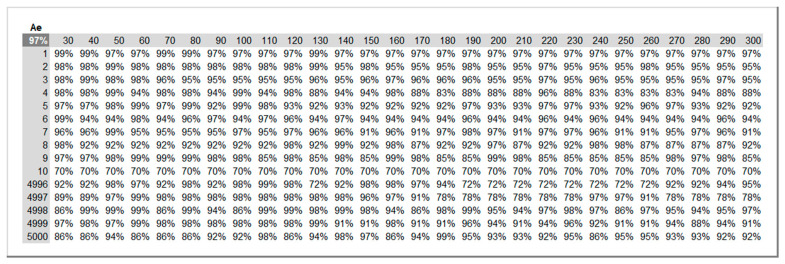
Equipment availability Ae across 5000 runs for inspection periods ΔT = 30 to 300 h.

A Monte Carlo simulation method is called from the “Simulations” sheet for each *KPI*: total maintenance costs, UC, and equipment availability Ae. The number R of runs and the set of inspections periods ΔT to simulate are defined in the “Inputs” sheet and automatically updated in this sheet. The simulation is conducted by taking advantage of the Excel “Data Table” feature as suggested by the discipline of probability management. Its advantages over running a VBA procedure are two-fold: it is much faster, and it allows recording and auditing each result. Figure 10 and Figure 11 illustrate excerpts of the results on the two *KPIs* for R = 5000 and for inspection periods ΔT ranging from 30 to 300 h in increments of 10 h.

**Figure 12:**
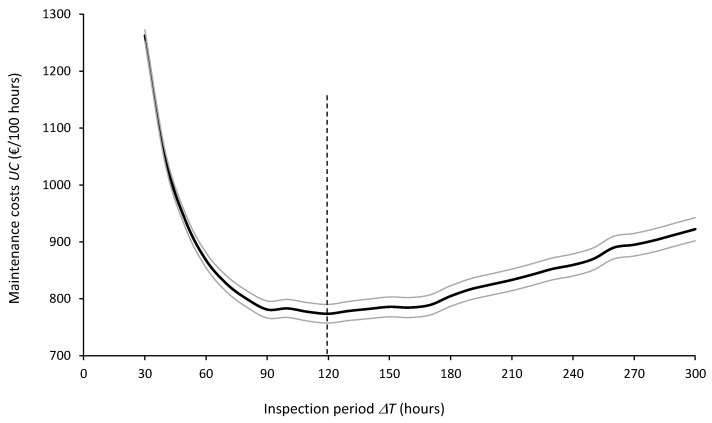
Expected values (thick central line) and 95% confidence intervals (thin outer lines) of maintenance costs (UC) for inspection periods ΔT = 30 to 300 h computed from 5000 simulation runs.

**Figure 13:**
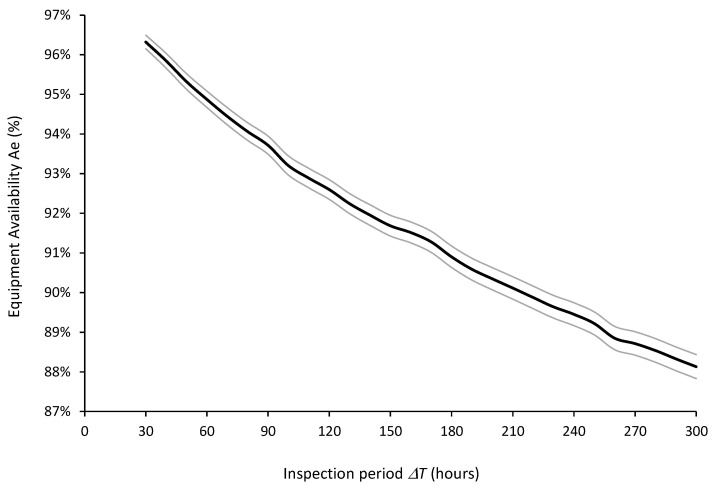
Expected values (thick central line) and 95% confidence intervals (thin outer lines) of equipment availability (Ae) for inspection periods ΔT = 30 to 300 h computed from 5000 simulation runs.

Figure 12 and Figure 13 show the expected values and 95% confidence intervals of maintenance costs (UC) and equipment availability (Ae), respectively, as a function of the inspection periods ΔT for R = 5000. On the one hand, it can be concluded from Figure 12 that the optimal inspection period from the point of view of minimizing maintenance costs is ΔT* = 120 h. On the other hand, from the point of view of maximizing equipment availability, Figure 13 clearly shows that the smaller the ΔT, the better the equipment availability.

### 4.5. Selecting the Optimal Inspection Period That Maximizes the Risk-Adjusted Overall Value to the Organization

Since it is not possible to define a ΔT that is optimal for both *KPIs*, the organization must choose the optimal inspection period ΔT* based on its intra-criterion and inter-criterion preferences. Since both *KPIs* are preferential and independent from the perspective of the organization, the additive multicriteria value model expressed in Equation (19) can be applied to evaluate the overall value $V\left(\Delta T\right)$ of each potential ΔT.

The value function $v\left(UC\right)$ for maintenance costs (UC) was assessed as linear by the organization, using the bisection method and defined as $v\left(UC\right)=100-UC/60$, based on Equation (20), with ${UC}^{-}$ = 6000 EUR/100 h and ${UC}^{+}$ = 0 EUR/100 h, considering the range of results obtained for UC in Section 4.4 (Figure 10).

Taking advantage of $v\left(UC\right)$ being linear, the value function $v\left(Ae\right)$ for equipment availability (Ae) and the weights $w_{UC}$ and $w_{Ae}$ for both *KPIs* were assessed by asking the organization’s decision-maker two questions: (a) How much is he (or she) willing to pay in EUR/100 h for improving equipment availability from 0% to 100%? (b) How much is he (or she) willing to pay in EUR/100 h for improving equipment availability from 0% to 50%? Based on his (or her) response of 9000 EUR/100 h to question (a), *KPI*’s weights were determined as $w_{UC}$ = 6000/(6000 + 9000) = 40% and $w_{Ae}$ = 9000/(6000 + 9000) = 60% by applying the special case of the tradeoff procedure described in Section 3.4. Likewise, based on a response of 7550 EUR/100 h to question (b), the value function for equipment availability was assessed as non-linear and defined exponentially as $v\left(Ae\right)=100\times \left[1-exp\left(-Ae/0.3\right)\right]/\left[1-exp\left(-1/0.3\right)\right]$, with ${Ae}^{-}$ = 0%, ${Ae}^{+}$ = 100%, and ρ = −0.3, determined by fitting Equation (21) to the points (0%, 0), (50%, 83.88), and (100%, 100) as described in Section 3.4 for the special case of the bisection method.

**Figure 14:**
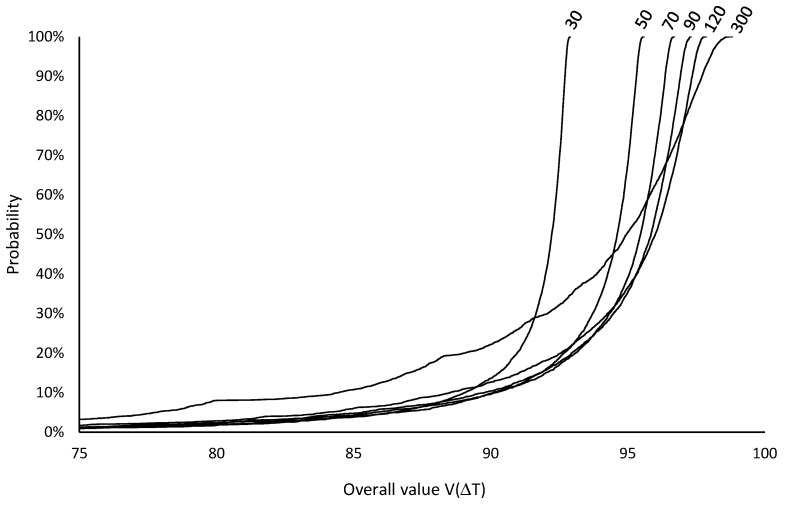
Cumulative risk profiles for the overall value $V\left(\Delta T\right)$ of inspection periods ΔT = 30, 50, 70, 90, 120, and 300 h (The x-axis was truncated before 75 to enhance clarity).

The overall values $V\left(\Delta T\right)$ for each inspection period ΔT, ranging from 30 to 300 h in increments of 10 h, were computed from five thousand simulation runs. These values were derived by combining the respective consequences on UC (Figure 10) and Ae (Figure 11) and then aggregating them using the additive model defined in Equation (19) with the specified weights and value functions. Figure 14 illustrates the respective cumulative risk profiles, as described in Section 3.4, for a subset of representative ΔT, showing that selecting the optimal inspection period based on (first order) stochastic dominance is not possible.

**Figure 15:**
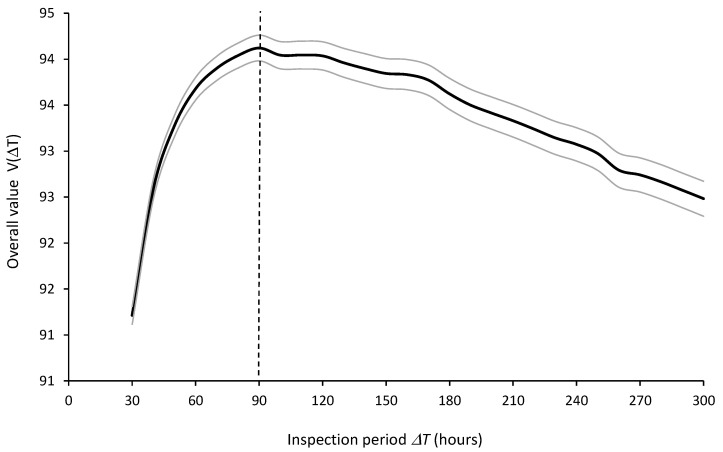
Expected values (thick central line) and 95% confidence intervals (thin outer lines) of overall value $V\left(\Delta T\right)$ for inspection periods ΔT = 30 to 300 h from 5000 simulation runs.

The optimal inspection period ΔT* should be selected based on the best expected value $\bar{V}\left(\Delta T\right)$. Figure 15 shows the expected values and 95% confidence intervals of the overall value $V\left(\Delta T\right)$ as a function of the inspection periods ΔT, computed from R = 5000 runs, indicating that the optimal inspection period is ΔT* = 90 h.

**Figure 16:**
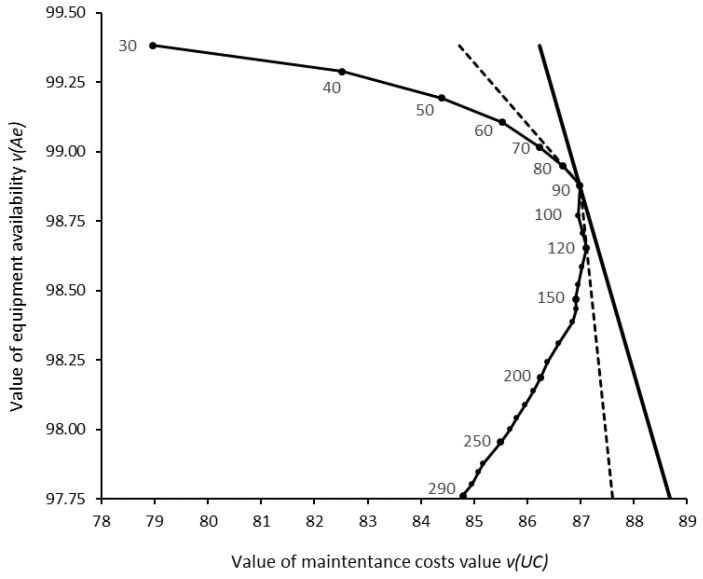
*KPI* value space. The dotted curve represents the values $v\left(UC\right)$ and $v\left(Ae\right)$ of inspection periods ΔT = 30 to 300 h. The solid line is the optimal $V\left(\Delta T*=90\right)$. The dashed lines represent $V\left(\Delta T*=80\right)$ and $V\left(\Delta T*=120\right)$.

A sensitivity analysis on the weights $w_{KPI}$ allowed to verify their impact on the selection of the optimal inspection period ΔT* from the set of inspection periods ΔT ranging from 30 to 300 h in increments of 10 h. It was found that the selection of ΔT* = 90 h is robust to changes in $w_{UC}$ between 18% and 65%, and, consequently, to changes in $w_{Ae}$ between 35% and 82%. These variations correspond to an interval between 3300 and 27,000 EUR/100 h (rather than 9000) regarding the amount that the organization is willing to pay for improving equipment availability from 0% to 100%, suggesting that the decision is indeed robust. Figure 16 plots the efficient frontier connecting the partial values $v\left(UC\right)$ and $v\left(Ae\right)$ of all evaluated inspection periods ΔT along with the isoline representing the optimal overall value $V\left(\Delta T*=90\right)=94.12$, with $w_{UC}$ = 40% and $w_{Ae}$ = 60%. Dashed lines represent the two iso-value lines for the weights that change the optimal inspection period ΔT* from 90 h to: (a) 80 h, when $w_{UC}$ = 18% and $w_{Ae}$ = 82% and (b) 120 h, when $w_{UC}$ = 65% and $w_{Ae}$ = 35%.

## 5. Discussion

This study presents a novel, integrated, and generic multimethodological approach for determining the optimal inspection period for protective sensors monitoring critical equipment. It considers multiple *KPIs* and the uncertainty associated with hidden failures in the system and the organization’s preferences. A case study illustrating the application of the proposed approach to a real-world maintenance problem via a DSS demonstrates its practical applicability and contributes to the limited literature on proactive maintenance modeling and optimization applied in practice [15]. The following sections summarize the main findings, implications, and limitations.

### 5.1. Findings

The modeling approach and DSS were developed to answer the five key research questions, identified in the Introduction, and commonly encountered in real-world applications of this maintenance problem. The results demonstrate that the proposed solution effectively addresses each specific research question as follows:

1. The use of three-parameter Weibull probability density functions, Monte Carlo simulation, and the principles of the probability management discipline enable an effective and auditable representation of the uncertainties related to failures in the equipment and sensors.
2. Discrete-event simulation modeling allows for accurate prediction of the consequences of these failures on multiple *KPIs*, considering varying sensor inspection periods, costs, time periods, and other input parameters.
3. Risk analysis and value-based multicriteria decision analysis methods facilitate risk-informed assessment of the overall value of different sensor inspection periods considering both their uncertain consequences and the preferences of the organization on multiple conflicting organizational *KPIs*, namely by representing the intertemporal, intra-criterion, and inter-criteria preferences of the organization through discount rate, value functions, and weights of the *KPIs*, respectively.
4. Risk-informed multicriteria value optimization methods, namely stochastic dominance and/or expected value maximization, provide a sound criterion for determining the optimal inspection period, while sensitivity analysis allows assessing the robustness of the optimal decision against the uncertainty of the organization’s preferences.
5. Finally, organizational buy-in and the successful implementation of maintenance decisions recommended by a decision support model require not only the scientific rigor and integration of the underlying approach, but also key success factors related to the DSS, such as its transparency, openness, understandability, user-friendliness, and auditability. These qualities were ensured by developing a prototype in Microsoft Excel, utilizing only built-in functions and taking advantage of the principles of probability management.

This research addresses a significant gap in the maintenance literature regarding the need for integrated methodologies that combine quantitative and qualitative data, uncertainty and risk analysis, and organizational preferences across multiple *KPIs*.

The proposed approach is generic, enabling it to accommodate different parameters and integrate additional assumptions, such as incorporating new *KPIs*, using alternative probability distributions for modeling failure uncertainty, or applying other MCVM methods for determining intra-criterion and/or inter-criteria preferences.

### 5.2. Implications

The proposed framework contributes to the state-of-the-art by providing a practical and robust approach for optimizing inspection scheduling for protective sensors monitoring critical equipment subject to uncertain hidden failures. In contrast to the traditional maintenance models, which often rely on simplified assumptions and deterministic approaches, this framework explicitly accounts for the uncertainty associated with sensor and equipment failures as well as organizational preferences, aligning with recommendations for more risk-informed multicriteria decision-making in industrial maintenance.

Another key contribution to practice is the development and provision of an open-access DSS in Excel, increasing transparency, comprehensibility, ease of use, and making it valuable both practical decision-making and educational purposes.

### 5.3. Limitations

The main limitations of the results include the following. The case study focused on a single protection sensor in a specific utility context, which limits generalization. The computational time required to run thousands of simulations can be significant, although using the Table Data feature from Excel instead of VBA macros reduces execution from hours to minutes. Another limitation regards the comparison of discrete inspection periods only, which might miss a better inspection period compared to the continuous case. One solution to this limitation comprises running the same problem again with a set of less evenly spaced inspection periods around the previous optimal inspection period. For instance, in the presented case study, running the DSS again with the optimization input parameters set with a minimum of 80 h, a maximum of 100 h, and a step of 1 h. Minor limitations, which can be easily addressed by model refinement, include modeling sensor inspection time if the sensor is not available to perform its protecting function while the inspection is performed; and probabilistically modeling the duration and/or costs of other maintenance tasks, namely regarding inspection, repair, and reactivation. Modeling assumptions such as perfect inspections, independent failure processes, and Weibull-distributed failure times may not fully capture real system complexities. Imperfect inspections or correlated failures could alter optimal intervals and risk profiles, and alternative distributions may be more suitable in some systems. These factors do not invalidate the framework but should be considered when generalizing the results.

## 6. Conclusions

This research addresses the problem of determining the optimal inspection period for protective sensors subject to hidden failures. The proposed approach combines simulation, optimization, and multicriteria decision analysis to support this complex decision.

The key finding is that a robust optimal inspection period can be identified by jointly modeling the randomness of failures and the organization’s preferences and risk attitude. In the case study, the period of 90 h represented the best compromise between cost and availability. This result provides an objective and auditable basis for maintenance policies, with a direct impact on reducing unplanned downtime and increasing operational reliability, which are central goals of proactive maintenance.

The proposed approach addresses the underlying research questions required to tackle this maintenance problem by: (a) modeling failure uncertainties using probability density functions, Monte Carlo simulation, and probability management: (b) predicting failure consequences on *KPIs* through discrete-event simulation; (c) comparing the overall value of alternative inspection periods using multicriteria value measurement and risk analysis methods; (d) determining the optimal inspection period considering uncertainties and preferences by stochastic dominance, expected value maximization, and sensitivity analysis; and (e) developing a user-friendly decision support system for practical implementation.

The developed framework and decision support system empower organizations to make risk-informed multicriteria decisions regarding sensor inspection schedules, leading to optimized industrial maintenance. This translates to reduced downtime, lower maintenance costs, and improved overall equipment effectiveness. The proposed integrated approach represents an advancement over traditional methods that often rely on simplified assumptions and single-criterion optimization. The methodology is readily applicable across various industries reliant on critical equipment with protective sensors, such as power generation, manufacturing, and transportation. By adopting this approach, organizations can enhance their proactive maintenance practices and improve operational efficiency.

Although the case study demonstrates the feasibility and usefulness of the proposed framework, additional empirical validation in real operational environments remains an important direction for future research. Implementing the optimized inspection schedule in practice and monitoring the resulting performance over time would provide evidence of real-world benefits and support the continuous improvement of the model.

Future research should explore the integration of real-time condition monitoring data into the proposed approach, allowing for even more dynamic and adaptive inspection schedules. Further investigation into the scalability of the framework for modeling complex networks with multiple protective sensors interconnected with critical equipment can also offer valuable insights. Two sensors can be used either as a protective function, or one can replace the other while it is being repaired, providing redundancy but with additional costs. Additionally, exploring the relevance of modeling non-neutral risk attitudes on the optimal inspection period could be investigated. We encourage researchers and practitioners to adopt and adapt this framework to their specific contexts, contributing to the advancement of proactive maintenance practices and improved asset management.

## Data Availability

The original contributions presented in this study are included in the article and Supplementary Material. Further inquiries can be directed to the corresponding author.

## Supplementary Materials

The following supporting information can be downloaded at: https://www.mdpi.com/article/10.3390/s26010213/s1, Prototype Excel application mentioned in the Case Study.

## Abbreviations

The following abbreviations are used in this manuscript:

DSS	Decision support system
*KPI*	Key performance indicator
MCVM	Multicriteria value measurement methods
MTBF	Mean time between failures
MTTF	Mean time to failure
MTTR	Mean time to repair
RCM	Reliability centered maintenance
VBA	Visual basic for applications
